# Examining sleep health and its associations with technology use among older adults in Sweden: insights from a population-based study

**DOI:** 10.1186/s12889-025-23894-8

**Published:** 2025-08-22

**Authors:** Sarah Nauman Ghazi, Anders Behrens, Johan Sanmartin Berglund, Jessica Berner, Peter Anderberg

**Affiliations:** Department of Health, Blekinge Institute of Technology, Valhallavägen, Karlskrona, 371 41 Blekinge Sweden

**Keywords:** Sleep health, Technology use, Older adults, SNAC-B, Gerontechnology

## Abstract

**Introduction:**

Exploring the association between technology use and sleep health in older adults is important as digital engagement becomes integrated into society.

**Objective:**

This study aimed to examine sleep health and its association with technology use in a population-based cohort of 60 years and older.

**Methods:**

This cross-sectional, population-based study (2023) included 436 older adults from the Swedish National Study on Aging and Care, Blekinge (SNAC-B) population. These participants were sent questionnaires about their sleep, internet usage, Digital Social Participation (DSP), Technology Anxiety (TA), Technology Enthusiasm (TE), and use of information and communication technology. We used a multidimensional instrument, SATED, to measure sleep health. In this study, we conducted statistical analyses using the chi2 test, T-test, Pearson correlation, and backward linear and logistic regression.

**Results:**

Our study found that older adults (60 years+) have a mean sleep health score of 7.40 (SD = 2.03). TE ($r = 0.18$, $p < 0.001$) and DSP ($r = 0.14$, $p = 0.004$) were positively associated with better sleep health, while TA ($r = -0.15$, $p = 0.003$) was negatively associated. Frequent internet users(M = 7.6) and engaging with screens before bedtime (M = 7.7) had higher sleep health scores compared to non-frequent users (M = 6.90, $p = 0.002$) and none or seldom engagement with screens before bedtime (M = 7.10, $p= 0.003$) respectively. Linear regression showed TE positively associated ($\beta$ = 0.241, $p=0.012$) while TA negatively associated ($\beta$ = -0.220, $p=0.029$) with sleep health. DSP was found to be a predictor of better satisfaction (OR: 1.32, $p= 0.009$), efficiency (OR: 1.16, $p=0.026$), and duration of sleep (OR:1.16, $p= 0.042$). Lower TA predicted better satisfaction (OR: 0.81, $p=0.04$), timing (OR: 0.74, $p=0.04$), and efficiency (OR:0.78, $p=0.01$) of sleep. Older adults who use technology one hour before sleep have better sleep timing (OR: 3.003, $p=0.002$), while those who do use mobile phones with a screen during the awake period after sleep onset have poor sleep timing (OR:0.016, $p=0.002$).

**Conclusions:**

DSP and TE support better sleep health, while TA negatively impacts sleep satisfaction, timing, and efficiency. Encouraging positive digital engagement and minimizing technology-related stress may promote healthier sleep in older adults.

**Supplementary Information:**

The online version contains supplementary material available at 10.1186/s12889-025-23894-8.

## Introduction

Sleep health is an important component of comprehensive health and overall well-being [1]. Sleep health had usually been conceptualized with sleep disturbances [2, 3] until Buysse et al. provided a new theoretical framework for comprehending sleep health, as “a multidimensional pattern of sleep-wakefulness, adapted to individual, social, and environmental demands, that promotes physical and mental well-being” [4]. Poor sleep health is associated with frailty [5], poor cognition [6, 7], and cardiovascular disorders [8]. While sleep efficiency and sleep onset may change with age [9], older adults can continue to maintain good sleep health by fostering regular sleep routines [10], achieving sufficient sleep duration, and lifestyle changes [11]. In a study, it was found that when comorbidity and illnesses are controlled, older adults are less likely to report sleep disturbances [12]. The perspective regarding the causal relationship between aging and sleep often leads to normalizing these disturbances, which are inevitable and non-modifiable to changes. This viewpoint can be limiting as it may prevent older adults and healthcare providers from engaging in solutions that can manage or alleviate these sleep disturbances. Sleep disturbances are not a definite part of the aging process [13, 14], and on the individual and societal level, there might be modifiable factors affecting sleep health.

Living in a digital society enables rapid information flow through global networks [15], and 24/7 connectedness to society through technology can significantly impact sleep health. From smartphones, tablets, and laptops to wearable and non-wearable devices, digital technology now infiltrates almost every aspect of modern living. The presence and usage of screen-based devices have been linked to delayed sleep onset, reduced sleep duration, and poorer sleep quality [16]. Technology-related attitudes play an important role in the digital world. Technology Anxiety (TA) describes the apprehension, or worry, some individuals feel when using everyday information-and-communication technologies [17]. TA is often linked to stress [18] and increased psychological vulnerability and lowered self-esteem [19, 20]. By contrast, Technology Enthusiasm (TE) reflects a curious and generally positive disposition toward adopting new devices and applications [17]. Technology-enthusiastic users might spend more time interacting with screens, particularly in the evening, thereby increasing exposure to stimulating content [21]. At the same time, TE can encourage the uptake of sleep-tracking apps and other self-monitoring tools that may promote healthier habits [22]. Digital social participation, although significant in maintaining psychological health in older adults [23], can impact sleep health by contributing to sleep disturbances due to increased engagement in social media activities [24]. In adults, higher usage of smartphones is associated with reduced sleep duration and lower sleep efficiency [25]. In another study comparing the younger population with older adults, it was found that using mobile phones before sleep leads to earlier waking times and reduced duration of sleep among older participants, specifically those aged 60.15 years and older and 66.4 years and older, respectively [26].

On the other hand, the integration of technology influences our social interactions, behaviors, and health [27]. Digital technologies can also support healthy sleep behaviors through sleep monitoring and improvement tools [28] and awareness of modifying sleep health on the internet [29]. In one study, the frequency of technology use significantly improved Chinese older adults’ health, including sleep [30]. The term used to describe factors related to digital technology use is digital determinants, developed and recognized by The World Health Organization (WHO). They are suggested as a subset of social determinants that include digital factors such as digital literacy, ease of use, and interactivity, among others, and their influence on health outcomes [31]. According to Chidambaram et al., there are several dimensions of digital health, such as Interactivity, Digital accessibility, Digital literacy, and Technology Personalization, among others. Digital technology has also been described as “super social determinants of health”, capable of addressing all other social determinants of health [32]. These digital determinants can play an important role in addressing various factors affecting sleep health.

Sleep health can be influenced by different circumstances, varying needs and behaviors, and diverse environments. A socio-ecological model of sleep has been proposed in which the determinants lie on three levels i.e., Individual, Social, and Societal. Digital determinants lie at the individual and societal levels. Understanding these determinants will help older adults modify these factors to support sleep health [33]. There are several studies looking at the associations of the use of the internet, screens, and televisions with sleep, but there is no study, in our current knowledge, that looks into these associations in terms of comprehensive digital determinants that are associated with sleep health and its dimensions in older adults. Internet usage among older adults in Sweden has risen significantly, with 85% online and 69% using it daily. Among those aged 65 to 75, 94% are internet users, and 82% access it daily. For those aged 75 and above, 72% use the internet, with 52% online every day [34]. This increasing use has also led to increased digital social participation [23, 35]. Additionally, older adults also utilize technology to monitor their health and maintain an active lifestyle [36]. Despite this, the studies that measure the association of technology and sleep mostly focus on younger populations rather than older adults [37–40]. Furthermore, multidimensional sleep health has yet to be investigated among community-dwelling older adults in Sweden, leaving an important evidence gap in our understanding of demographic patterns and determinants of sleep health in this population. Therefore, the aim of this study was twofold: (1) to examine multidimensional sleep health in a population-based sample of older adults in Sweden, and (2) to investigate the association of sleep health with technology use in a population-based cohort of 60 years and older in Sweden.

## Methods

This study is based on data from the Swedish National Study on Aging and Care (SNAC), a longitudinal population-based study that collects health and lifestyle data on older adults (60 years and above) across multiple regions in Sweden. In SNAC, the sample size was determined based on approximately 10% of each cohort population i.e. from Kungsholmen, Nordanstig, Skåne, and Blekinge. Data is gathered every three years through questionnaires, interviews, and medical examinations. Every sixth year, individuals who are 60 years old are added to the study. This research aims to obtain information on the participants’ health, psychological state, functionality, social circumstances, and care requirements [41]. SNAC-Blekinge (SNAC-B) is one of the regional cohorts, specifically focusing on older adults in Blekinge, Sweden. Participants in this regional cohort represent a random sample of the population aged 60 and above years old and living in a municipal area in the southeast of Sweden (Blekinge) with a population of approximately 64,000 [42]. For the present analysis, we included all SNAC-B participants who completed the sleep-health and digital-technology survey in 2023. This survey was sent to all SNAC-B participants aged 60+ to assess sleep health and technology use, excluding those living in dementia care homes or special care homes and people with severe cognitive disorders.

### Data collection

**Fig. 1:**
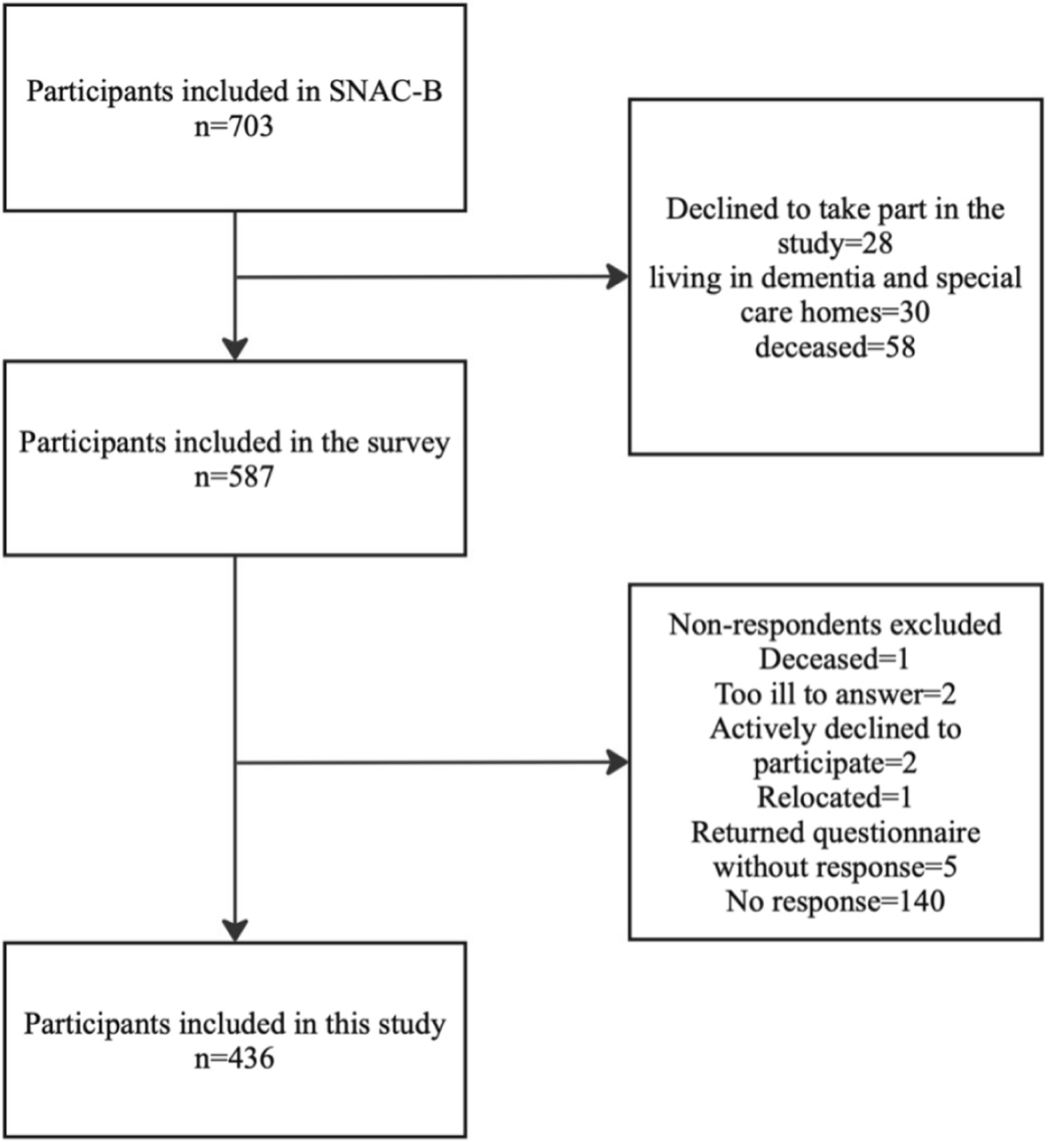
Flow diagram for the selection of the participants

Out of the total SNAC-B population ($N = 703$), questionnaires were sent to 587 participants aged 60 and above, through post and email. Participants were informed about the nature of the questions and their right not to respond. The questionnaire was accompanied by instructions on how to return the completed questionnaire, and the contact information for seeking help in case any clarifications were needed was also provided. The participants who did not respond the first time were also sent a reminder. Out of 587, 436 (74.2%) participants responded. The non-respondents ($n = 151$) consisted of 1 deceased person, 2 individuals who were too ill to answer, 2 who actively declined to participate, 1 who had relocated, and 5 who returned the questionnaire without any response. The remaining 140 individuals did not return the questionnaire (See Fig. 1). Among the total non-respondents, 80 were females (53.69%), while 69 (46.30%) were males. The mean age of non-respondents was 83.3 (SD = 7.35). The oldest old (85+ years) had the most non-respondents ($n = 80$, 46%).

### Measures

The questionnaire included instruments such as SATED for sleep health (See “Sleep health variables” section), DSP, and TechPH dimensions TA and TE (“Digital determinants variables” section), which were used after seeking the permission of the creators of these instruments, and they have been used in published studies before. Other questions regarding technology use for this study were developed for this study and have not been published before. The survey questions were pilot-tested with four older adults to ensure clarity and ease of understanding. After completing the survey, they were interviewed about their comprehension of the questions and asked if there was anything they would like to change. The survey was edited accordingly to increase the reliability of the survey. The variables included in the study are:

#### Sociodemographic variables

The included variables were age, gender, living arrangement, and education. The age was categorized into three groups: young-old (65 - 74 years), middle-old (75 - 84 years), and oldest-old (85+ years). Gender was categorized into Male and Female. The education variable was categorized according to the Swedish old education system relevant to our participants’ age into three groups: Primary (Incomplete primary/elementary school, up to primary/elementary school), Secondary (Secondary school, high school, vocational training), and higher (University /college and further) levels of education. The participants were also asked about their living arrangements, whether they lived alone or with someone. Sleep disorders were assessed using a self-reported diagnostic question, asking participants whether they had been diagnosed with a sleep disorder by a healthcare professional (Yes/No). Those who answered Yes were asked to specify their diagnosis in an open-ended format. Examples of the disorders were provided as sleep apnoea, narcolepsy, restless leg syndrome, and others.

#### Sleep health variables

**Fig. 2:**
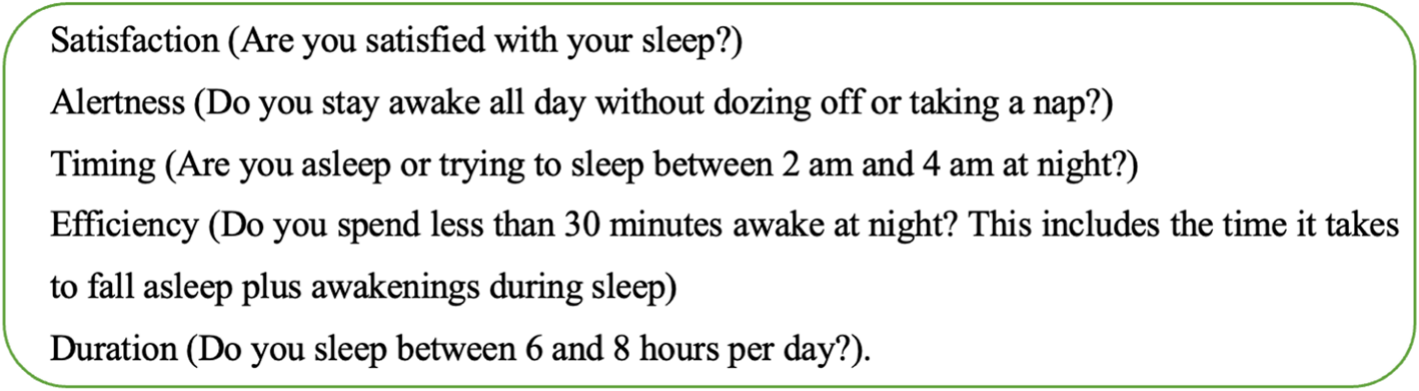
SATED Questionaire

Sleep health was assessed with a validated instrument, SATED v1.0 [4, 43]. SATED is a multidimensional and comprehensive measure of sleep health, which is quick to deploy in large surveys as it contains only five questions, unlike The Pittsburgh Sleep Quality Index (PSQI), which contains nineteen questions [44]. After obtaining permission from the University of Pittsburgh, which holds the copyright for the SATED instrument, it was translated from English to Swedish by two native speakers for the participants. The translated instrument also went through a pilot test where we asked four older adults to fill out the instrument and asked them follow-up questions about the comprehension of the questions in the instrument. The SATED questionnaire has a 3-point Likert scale (Never (1), Sometimes (2), and Always (3)) and consists of sleep health dimensions Satisfaction, Alertness, Timing, Efficiency and Duration. Specific questions for each of these dimensions are seen in Fig. 2. A Total Sleep Health Score (0-10) was calculated by adding the dimensions’ score, with higher scores suggesting good sleep health [4]. The sleep health dimensions we dichotomized for logistic regression with Never and Sometimes coded as 0 and always coded as 1. The SATED questionnaire showed adequate internal consistency (Cronbach’s $\alpha$ = 0.74).

#### Digital determinants variables

Based on the four dimensions of digital determinants of health [31], the SNAC survey included several variables, which are the factors or determinants related to technology usage. The four digital determinants were Digital accessibility, Interactivity, Technology personalization, and Technology acceptance. Digital accessibility was assessed through internet usage (Do you use the Internet? Yes/No) and frequency of use (Never or seldom, Often). Interactivity was assessed using several measures: the use of technology with a screen one hour before sleep (Never/Seldom, Always) and the use of a mobile phone with a screen if awake at night. Additionally, perceptions of the benefits of online social interactions were measured using an instrument called Digital Social Participation (DSP) [45]. It is a questionnaire consisting of six questions regarding social connectedness and social relations. The total DSP score of the participants ranges from 6-30. In this study, the total social DSP score is presented as a mean score (1–5) to depict the weighted average of each item in the total DSP score. The higher the score the better the DSP. Technology personalization was measured through questions about the use of technology to monitor sleep and the use of technology to improve your sleep. Technology acceptance came under the umbrella of digital literacy and was assessed using two dimensions of the TechPH instrument: Technology Anxiety (TA) and Technology Enthusiasm (TE) [17]. These two dimensions consisted of six questions, three questions in each. The scores for both of them were calculated separately. Higher scores represented higher on either scale. DSP and TechPH are validated instruments while other questions regarding digital determinants mentioned above were designed to explore additional aspects of technology use. The internal consistency (Cronbach’s $\alpha$) of DSP was 0.50, TE was 0.55 and TA was 0.53.

#### Statistical analysis

The normality of the data was assessed with Q-Q plots and skewness or kurtosis [46]. Continuous variables were summarised with mean and standard deviation, and the categorical variables with frequency (n) and the proportion percentage (%). Missing data was excluded based on listwise deletion. The sleep health differences based on gender and age groups were identified using cross-tabulations and the Chi2 test as the statistical test. This was performed because there is no study in our knowledge that looks into the association of gender and age group with the five sleep health dimensions. An independent samples t-test was conducted to explore the difference in the mean sleep health scores across different digital determinants with categories. Pearson correlation was performed to examine the association of digital social participation, technology anxiety, and technology enthusiasm with the total sleep score. Backward linear regression with total sleep health score as the dependent variable and all the independent variables mentioned in “Sociodemographic variables”, “Digital determinants variables” were employed to identify the most relevant predictors of sleep health. Backward logistic regression was performed separately for each of the five sleep health component variables to determine which independent variables affect the five dimensions of sleep health. To check for robustness of both the regressions, we re-estimated a full multivariable model with all predictors forced. For each binary SATED dimension, we fitted the corresponding full model alongside the backward specification. All sensitivity results are provided in the Supplementary File. The backward logistic and linear regression method was chosen as it allows for the most significant predictors of sleep health to come up by systematically eliminating non-significant variables to provide concise final models that focus on the primary factors that influence sleep health. Results from the linear regression are presented as a Beta coefficient ($\beta$), Significance, and Confidence Intervals (CI). Logistic regression models are presented as Odds Ratios (OR) with 95% Confidence Intervals (CI). An OR >1 indicates an increased likelihood of the outcome, while an OR <1 suggests a decreased likelihood.

The independent variables in the “Sociodemographic variables” and “Digital determinants variables” sections remained consistent across all models. All the ’digital determinants variables’ apart from DSP and TechPH were converted to binary for the practical reason of doing an independent sample T-Test. The variables such as Internet user, technology to monitor sleep, and technology to improve sleep were already binary. The variables frequency of technology use, Use of technology with a screen one hour before sleep, and Mobile phone with a screen during the awake period after sleep onset were converted to binary with the ’seldom or never’ option as 0 while ’often’ as 1. The use of binary variables was primarily to ensure homogeneity and interpretability of groups in a straightforward manner. However, we acknowledge that this might reduce granularity in the data and can limit statistical power. The statistical significance value was set to 0.05. The data was analyzed using IBM SPSS (Version 29.0.1.0 (171)).

## Results

The demographics of the sample population are shown in Table 1. The total sample population was $N= 436$, of which 232 (53.2%) were female. The mean age in the sample was 78 years (SD = 7.7, Range = 61 - 100). Almost half of the participants ($n = 187$, 45.4%) had a secondary education level and lived together with someone ($n = 281$, 65%). In our sample, most of the participants were internet users ($n = 349$, 80%). The mean Total Sleep Health Score of the sample was 7.40 (SD = 2.03; Range = 0 - 10). In our sample, 10.4% ($n = 43$) of participants reported a diagnosed sleep disorder.

**Table 1:** Descriptive Statistics of all the variables used ($N=436$)

Variables	Categories	Frequency (%)	Mean Sleep Health score (SD)
**Age groups**	60-74 years	150 (34.4)	7.75 (1.98)
	75-84 years	193 (44.3)	7.34 (2.06)
	85+ years	93 (21.3)	6.89 (1.94)
**Gender**	Men	204 (46.8)	7.57 (1.87)
	Women	232 (53.2)	7.23 (2.15)
**Education**	Primary education	101 (24.5)	7.03 (1.86)
	Secondary education	187 (45.4)	7.19 (2.09)
	Higher education	124 (30.1)	7.90 (2.03)
**Living status**	Alone	151 (35.0)	7.03 (2.2)
	Together with someone	281 (65.0)	7.58 (1.9)
**Internet User**	Not Internet user	87 (20.0)	6.90 (1.86)
	Internet user	349 (80.0)	7.50 (2.05)
**Frequency of technology use**	Seldom or never	128 (29.4)	6.89 (1.84)
	Often	308 (70.6)	7.59 (2.07)
**Use of technology with screen one hour before sleep**	Seldom or never	224 (52.5)	7.10 (2.14)
	Often	203 (47.5)	7.70 (1.85)
**Mobile phone with screen during awake period after sleep onset**	Seldom or never	403 (94.4)	7.42 (2.04)
	Often	24 (5.6)	6.86 (1.86)
**Do you use technology to monitor sleep?**	No	400 (91.7)	7.40 (2.03)
	Yes	21 (4.8)	7.44 (2.06)
**Do you use technology to improve sleep?**	No	339 (77.8)	7.44 (2.06)
	Yes	97 (22.2)	7.20 (1.9)
**Sleep disorder**	No	371 (89.6)	7.48 (1.99)
	Yes	43 (10.4)	7.00 (2.28)
**Satisfaction of sleep**	Never or sometimes	174 (41.4)	5.89 (1.95)
	Always	246 (58.6)	8.45 (1.28)
**Alertness during the day**	Never or sometimes	200 (47.5)	6.55 (1.95)
	Always	221 (52.5)	8.13 (1.80)
**Timing of sleep**	Never or sometimes	63 (15.1)	4.77 (2.06)
	Always	355 (84.9)	7.86 (1.63)
**Efficiency of sleep**	Never or sometimes	261 (62.9)	6.65 (1.91)
	Always	154 (37.1)	8.64 (1.57)
**Duration of sleep**	Never or sometimes	127 (30.4)	5.29 (1.86)
	Always	291 (69.6)	8.29 (1.31)
	**Mean**	**Standard deviation**	**Range**
**Total Sleep Health Score**	7.40	2.03	1-10
**Digital Social Participation**	2.64	1.69	1-5
**Technology Anxiety**	3.3	1.1	1-5
**Technology Enthusiasm**	2.9	1.1	1-5

**Fig. 3:**
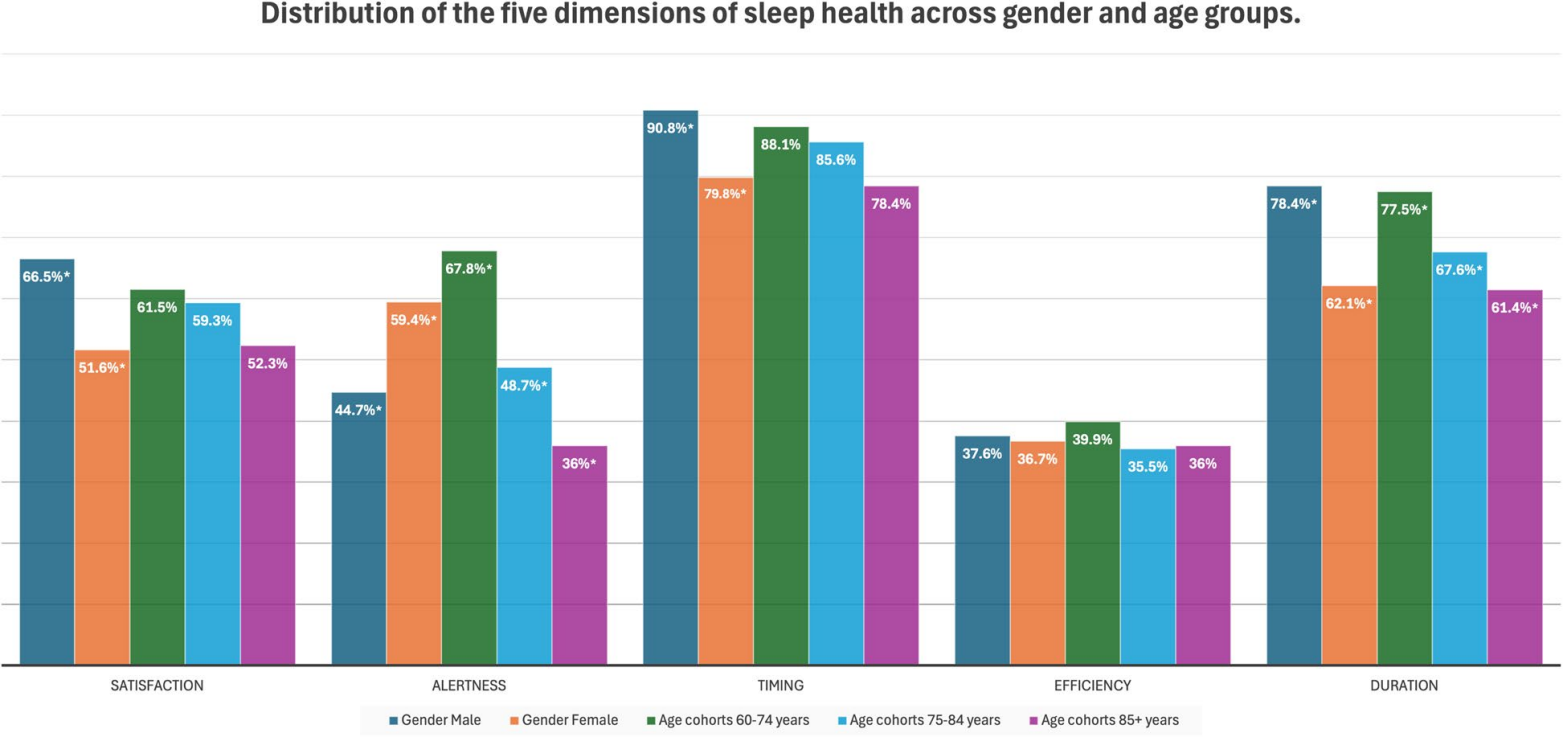
Distribution of the ’Always’ category in the five dimensions of sleep health across gender and age. The x-axis represents the five sleep health dimensions, while the y-axis shows the percentage of participants reporting “Always” in each dimension. Significant values are marked with an asterisk (*)

In this study, males and younger age groups (60 - 74 years) had better mean sleep scores than females and those with higher age groups (75+), respectively (Table 1). Sleep health dimensions stratified by age and gender are displayed in Fig. 3. When examining the difference in sleep health dimensions in gender, males had significantly better satisfaction ($p = 0.002$), timing ($p = 0.002$), and duration ($p<0.001$) of sleep, while females had significantly better alertness ($p = 0.003$) during the day. No gender differences were detected for efficiency. Significant associations were found with age groups and sleep health dimensions of alertness ($p<0.001$) and duration ($p = 0.025$). Examining the age groups, the younger age group (60 - 74 years) had better alertness and duration of sleep than the oldest old (85+ years) age group. For a detailed breakdown of the dimensions of sleep health across gender and age groups refer to Table 2.

**Table 2:** Difference in Sleep health dimensions across gender and age. *P*-Value: Chi2 test Significant values are marked with a *. Cut-off point 0.05

	Male	Female	*P*-Value	60-74 years	75-84 years	85+ years	*P*-Value
	n (%)	n (%)		n (%)	n (%)	n (%)	
**Satisfaction**			0.002*				0.369
0 Never/sometimes	66 (33.5)	108 (48.4)		55 (38.5)	77 (40.7)	42 (47.7)	
1 Always	131 (66.5)	115 (51.6)		88 (61.5)	112 (59.3)	46 (52.3)	
**Alertness**			0.003*				$<0.001$*
0 Never/sometimes	109 (55.3)	91 (40.6)		46 (32.2)	97 (51.3)	57 (64.0)	
1 Always	88 (44.7)	133 (59.4)		97 (67.8)	92 (48.7)	32 (36.0)	
**Timing**			0.002*				0.128
0 Never/sometimes	18 (9.2)	45 (20.2)		17 (11.9)	27 (14.4)	19 (21.6)	
1 Always	177 (90.8)	178 (79.8)		126 (88.1)	160 (85.6)	69 (78.4)	
**Efficiency**			0.855				0.699
0 Never/sometimes	123 (62.4)	138 (63.3)		86 (60.1)	120 (64.5)	55 (64.0)	
1 Always	74 (37.6)	80 (36.7)		57 (39.9)	66 (35.5)	31 (36.0)	
**Duration**			$<0.001$*				0.025*
0 Never/sometimes	42 (21.6)	85 (37.9)		32 (22.5)	61 (32.4)	34 (38.6)	
1 Always	152 (78.4)	139 (62.1)		110 (77.5)	127 (67.6)	54 (61.4)	

**Fig. 4:**
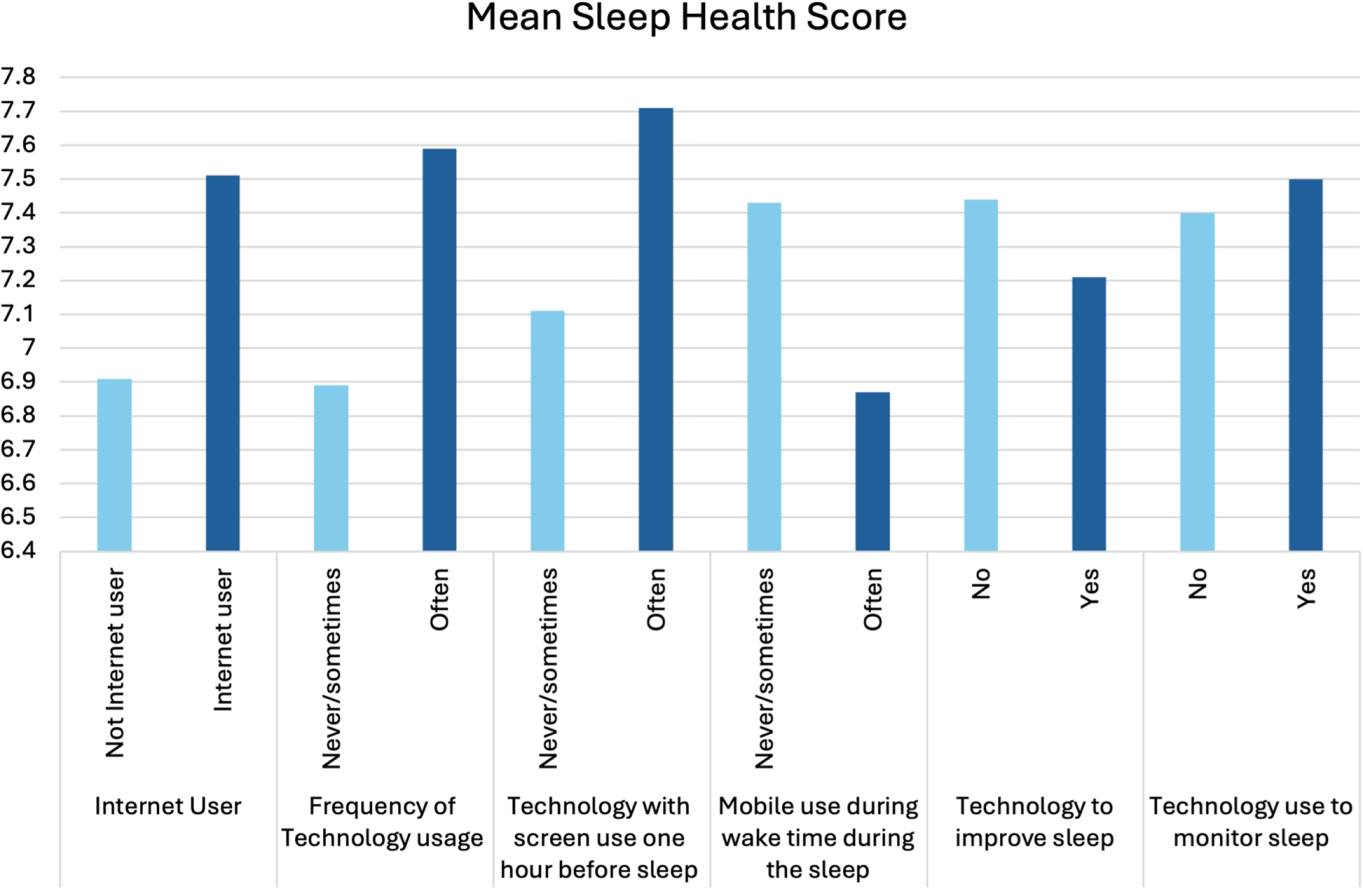
Digital determinants and Mean total sleep health score. The x-axis represents the different digital determinants like internet users or frequency of technology usage. The y-axis shows the mean total sleep health score for each category of the determinant

Table 3 presents all the digital determinants and their association with total sleep health score. The variables of TE ($r= 0.177$, $p<0.001$), TA ($r= 0.149$, $p=0.003$), and DSP ($r= 0.144$, $p=0.004$) were significantly correlated with the total sleep health score. TE and DSP were positively correlated, while TA was negatively correlated. Further, significant associations were seen between total sleep health score and other variables such as internet users ($p = 0.021$), frequency of technology usage ($p = 0.002$), and use of technology with a screen one hour before sleep ($p = 0.003$). Internet users had a mean total sleep health score of 7.5, while those who did not use the internet had a score of 6.9. Older adults who often use technology had better sleep health (mean 7.59) than those who never or seldom use technology (mean 6.89). Those older adults who use technology with a screen one hour before sleep had better sleep health scores (7.7) than those who do not (7.1). This difference has a small to moderate effect size (Cohen’s D = -0.298) but was still statistically significant (*P* value=0.003). Figure 4 shows a graphical representation of the Total Sleep Health Score and different digital determinants.

**Table 3:** Association of Digital Determinants with Total Sleep Health.$^a$-Pearson correlation $^b$-T-test. Significant values are marked with an asterisk *

Variables	Correlation Coefficient/t value	*P* value
Technology Enthusiasm$^a$	0.177	$<0.001$*
Technology Anxiety$^a$	-0.149	0.003*
Digital Social Participation$^a$	0.144	0.004*
Internet User$^b$	-2.312	0.021*
Frequency of Technology usage$^b$	-3.142	0.002*
Technology to improve sleep$^b$	0.945	0.345
Technology use to monitor sleep$^b$	-0.206	0.837
Technology with screen use one hour before sleep$^b$	-3.012	0.003*
Mobile use during wake time after sleep-onset$^b$	1.28	0.201

**Table 4:** Predictors of total sleep health score

Dependent variable	Independent variables in the model	Beta coefficient ($\boldsymbol{\upbeta}$)	Standard error (S.E)	Significance	Confidence interval	
					Lower bound	Upper bound
**Total sleep health score**	Education	0.281	0.150	0.062	-0.014	0.576
	Use of technology before sleep	0.398	0.215	0.064	-0.024	0.820
	Technology Enthusiasm	0.241	0.096	0.012*	0.053	0.429
	Technology Anxiety	-0.220	0.100	0.029*	-0.418	-0.023
	(Constant)	6.656	0.563	<0.001*	5.549	7.763

In our backward linear regression, Table 4, the final model after removing non-significant variables, identified significant predictors of sleep health scores. Education showed a positive association with sleep health but was marginally non-significant ($\beta$ = 0.281, $p = 0.062$). Using technology with screens one hour before sleep had a positive but non-significant effect ($\beta$ = 0.398, $p = 0.064$). Technology enthusiasm was positively associated with better sleep health ($\beta$ = 0.241, $p = 0.012$), while technology anxiety showed a significant negative association ($\beta$ = -0.220, $p = 0.029$).

Table 5 presents the findings regarding the effect of digital determinants on different dimensions of sleep health. All the regression models were statistically significant ($p < 0.05$). The logistic regressions revealed that females had lower odds of sleep satisfaction compared to males (OR: 0.49, $p=0.014$), controlling for other factors. Higher TA predicted lower sleep satisfaction (OR: 0.81, $p=0.04$). Increased (DSP) was associated with greater sleep satisfaction (OR: 1.32, $p = 0.009$). The presence of a sleep disorder significantly decreased the likelihood of sleep satisfaction ($p < 0.05$). The satisfaction model showed that being female, having TA, having lower DSP, and the presence of a sleep disorder were all significant predictors of less sleep satisfaction. For alertness, older adults in age groups old (74 - 85) and oldest (85+) reported lesser alertness during the day than younger age groups (60 - 74). Compared to primary education, those with higher education were more alert during the day. Our results for alertness also showed that using sleep monitoring technology was associated with lower daytime alertness (OR: 0.18, $p=0.006$). The alertness model thus showed that being higher in age, not being female, not having higher education, and monitoring one’s own sleep were significantly associated with less alertness. Females with TA (OR: 0.74, $p = 0.04$) and who use mobile phones with screens during awake periods after sleep onset (OR: 0.016, $p=0.002$) predicted poor sleep timing, while the use of technology with screens one hour before sleep predicted better sleep timing at night (OR: 3.003, $p=0.002$). The efficiency of sleep was associated with TA and DSP. TA predicted poor (OR: 0.78, $p = 0.01$), while DSP predicted better (OR: 1.16, $p = 0.026$) sleep efficiency. Duration of sleep was associated with being female and DSP. Females tend to have a shorter sleep duration than males, and those with DSP have a better sleep duration(OR: 1.16, $p = 0.042$).

Sensitivity analysis of linear regression showed that the adjusted R^2^ changed only from 0.064 in the backward solution to 0.060 (full) (Table S1). For each SATED dimension in logistic regressions, the full logistic models increased Nagelkerke R^2^ by $\le$ 0.03 relative to the backward models (Table S6). Effect directions and statistical significance for all technology-related predictors were unchanged, confirming that the primary findings are robust to model specification.

**Table 5:** Digital determinants predictive of 5 dimensions of sleep health

Dimensions of Sleep Health	Independent Variable	B	SE	Sig.	Odds Ratio	Confidence Interval		Significance of the Model	
						Lower	Upper	Chi2	Sig.
**Satisfaction**	Gender (Female)	-0.704	0.229	0.002	0.495	0.315	0.775	30.2	$<.001$
	Technology anxiety	-0.214	0.107	0.046	0.807	0.654	0.996		
	Digital Social Participation	0.278	0.106	0.009	1.320	1.071	1.626		
	Presence of sleep disorder	-0.778	0.354	0.028	0.460	0.230	0.920		
	Constant	1.573	0.482	0.001	4.819				
**Alertness**	Age Group (74-85)	-0.690	0.260	0.008	0.502	0.301	0.835	43.729	$<.001$
	Age Group (85+)	-0.963	0.335	0.004	0.382	0.198	0.736		
	Gender (Female)	0.651	0.225	0.004	1.918	1.234	2.981		
	Education (secondary)	0.345	0.296	0.244	1.413	0.790	2.525		
	Education (Higher)	0.807	0.328	0.014	2.241	1.177	4.266		
	Monitor sleep	-1.717	0.620	0.006	0.180	0.053	0.606		
	Constant	-0.428	0.375	0.254	0.652				
**Timing**	Gender (Female)	-1.033	0.347	0.003	0.356	0.180	0.703	39.544	$<.001$
	Technology Anxiety	-0.304	0.154	0.049	0.738	0.546	0.999		
	Use of technology before sleep	1.100	0.349	0.002	3.003	1.517	5.946		
	Mobile phone during awake	-1.839	0.592	0.002	0.159	0.050	0.507		
	Constant	3.265	0.700	$<.001$	26.175				
**Efficiency**	Technology anxiety	-0.243	0.104	0.019	0.784	0.640	0.961	12.12	0.007
	Digital Social Participation	0.152	0.068	0.026	1.164	1.018	1.331		
	Constant	-0.164	0.415	0.693	0.849				
**Duration**	Gender (Female)	-0.887	0.250	$<.001$	0.412	0.252	0.673	27.330	$<.001$
	Digital Social Participation	0.147	0.072	0.042	1.158	1.005	1.334		
	Constant	1.482	0.482	0.002	4.401				

## Discussion

We aimed to explore sleep health and investigate how technology use and other digital factors affect the subjective sleep health of older adults. There were several key findings in this study.

While exploring the sleep health of older adults in our population, we found gender differences in sleep health, with males generally experiencing better satisfaction, timing, and duration of sleep than females, who showed greater alertness during the day. Older adults aged 60 - 74 had better daytime alertness and duration of sleep (6 - 8 hours) in contrast to those over 85. We also found that digital factors such as TE and DSP are associated with better sleep health, while TA is associated with poor sleep health. We also identified an association between better sleep and being an internet user, using the internet often, and using technology with a screen one hour before sleep. The study also revealed that TA negatively impacts sleep satisfaction and efficiency. DSP positively influences sleep satisfaction, efficiency, and duration of sleep. The use of technology with a screen before sleep negatively affects sleep timing. Furthermore, monitoring sleep is negatively associated with alertness during the day.

The mean total sleep health score of the sample was 7.40, which, on a scale of 0 - 10, indicates good sleep health. The prevalence of even moderate sleep disturbances has been shown to be low among older adults in Sweden [47]. The higher sleep health scores in older adults compared to the younger population may be due to their adaptation to living with less sleep and complaining less, even in the presence of objective sleep disturbances [12]. Two studies from the United States [48] and Norway [49] also found that older adults had better sleep health, with higher mean scores compared to the younger population. Although there are biological changes and circadian blunting that might affect sleep [50], these findings suggest that sleep disturbances are not an inherent part of the aging process. Instead, various factors such as individual characteristics, social interactions, and societal influences impact sleep in older adults [33]. Another reason might be the difference between objective and subjective measures of sleep. Older adults often perceive their sleep health as better than what objective measures indicate [12]. Our results showed gender differences in sleep health dimensions which might be because women are less satisfied with their sleep and more likely to experience sleep problems [51]. However, women in this age group tend to be more in touch with their feelings and express them more openly [52], highlighting the need to objectively evaluate sleep health [53]. Additionally, compared to people 85 years old and above, older adults aged 60 - 74 have better daily alertness and a larger proportion sleep 6-8 hours at night. This difference in alertness with age may be attributed to cognitive functioning [54], physiological health and activity levels aligning with previous findings that show a decline in daytime alertness with age [55].

Our study highlighted that technology use has a positive association with self-reported sleep health. The relationship between technology use and sleep health may be influenced by the participant’s work experience, particularly if it involves frequent use of technology or the internet. Individuals with a background in technology-related fields may exhibit greater comfort and familiarity with digital tools, which could reduce anxiety and improve the quality of their technology engagement [56]. Frequent internet users, those with better DSP and higher TE, have better sleep health. DSP was found to be a predictor of satisfaction, efficiency, and duration of sleep. This may be because older adults who have increased participation in society have better sleep than those who do not [57]. DSP has been shown to be associated with higher subjective well-being and lower psychological distress by reducing loneliness and increasing social engagement in this population [23, 58, 59]. This effect of DSP on well-being might affect the sleep health of older adults.

Lower TA predicted better satisfaction, timing, and efficiency of sleep. These results might be confounded by general anxiety, which can disturb sleep in older adults [60]. TA may lead to stress and worry [18], and stress has been shown to have a negative effect on sleep [61], which might be the reason for our result. There are no specific studies, as per the knowledge of the authors, that directly measure the association of TA and TE on sleep.

Notably, older adults who use technology one hour before sleep have better sleep health, specifically better sleep timing. Conversely, those who do not use mobile phones with a screen during the awake period after sleep onset are also reported to have better timing of sleep. Previous studies have shown that bedtime use of technology is negatively associated with sleep parameters [26]. Blue light emitted from screens has often been linked to sleep disturbances because it suppresses melatonin, the hormone that promotes sleep [62]. However, a systematic review of five studies examining the impact of blue light on sleep revealed mixed results [63]. One study reported an increase in sleep quality, another indicated a decrease, while three studies found no significant change in sleep quality in the presence of blue light [63]. Another systematic review also suggests that blue light from screens has little effect on sleep [64]. The study found that screen brightness, even at its highest level of 80 lux, is far below the 500 lux required to impact sleep patterns. A review of 11 global studies showed that screen use delays sleep onset by only 9.9 minutes. These findings indicate that inconsistent sleep habits, rather than screen exposure, are the main contributors to sleep disturbances[64].

The systematic review also provided protective moderating factors that moderate the relationship between technology use and sleep, such as self-control, higher self-efficacy, or the type of content seen before bedtime [64]. Self-control is the ability to regulate our effective, cognitive, and behavioral response tendencies (or impulses), which is seen as an effortful and conscious process [65]. Self-efficacy is a generative capability that integrates cognitive, social, and behavioral skills into organized action. Technology use entails not just digital literacy but the ability to regulate engagement and maintain control over usage [66]. Higher self-efficacy and self-control can significantly improve one’s ability to limit technology use and help avoid delaying bedtime, thereby fostering improved sleep health [67, 68]. Our study findings align with this perspective, suggesting that technology use before sleep has a positive association with older adults. Most research on the relationship between technology and sleep focuses on younger adults and adolescents, where sleep displacement, bedtime procrastination, and nighttime arousal are commonly observed [39, 40, 69]. However, these findings may not be entirely applicable to older populations, who often interact with technology in different ways and may experience unique benefits from its use. Future research should investigate the specific types of content consumed, the various forms of technology utilized, and the strategies older adults use to manage their screen time. Technology might also facilitate sleep by diverting attention from stress, anxiety, or negative thoughts, which may otherwise prevent individuals from falling asleep [70, 71]. On the other hand, time spent with technology might also lead to later bedtimes and later sleep onset time, causing sleep displacement [37]. The real issue might not be the use of technology or screens but the ability to self-regulate time by using it later than the intended bedtime [64]. Moreover, older adults in Sweden have a high level of eHealth literacy [72], which correlates with more responsible and controlled use of technology for seeking online health information, leading to better health promotion behaviors, such as improved sleep habits and thus sleep quality [73, 74].

Our findings highlight associations between technology use and sleep health in older adults, where we accounted for key sociodemographic variables and the presence of sleep disorders that can influence technology usage and sleep health. However, the absence of additional health indicators and comprehensive measures of physical and cognitive health limits our ability to examine how these factors may moderate our results. Despite this, the observed associations suggest that technology-related factors (e.g., digital social participation, technology enthusiasm, technology anxiety) may play a role in older adults’ sleep health.

### Limitations and strengths of the study

One of the limitations of this study is that it is a cross-sectional study, which cannot establish the causality of the variables discussed. Furthermore, using self-reported sleep metrics can be challenging, especially in ensuring accuracy among older adults and those with some cognitive impairment. Self-reported sleep metrics might introduce recall bias (difficulty accurately remembering sleep times) and social desirability bias (reporting sleep patterns believed to be more acceptable). Future studies could benefit from incorporating objective sleep-monitoring technologies and objective measurement of technology to look into the association objectively. Another limitation is that the sample of this study is only from Blekinge, which is a small to mid-size town. Having a wider sample investigating the rural-urban Sweden population with a larger sample might give us different results for older adults or confirm our results, increasing the generalizability of the findings across different geographical contexts. Our study included only older adults who did not have cognitive decline or were very frail. Thus, there is a risk of bias when only healthy individuals are included. Furthermore, there might be several other factors or covariates, for example, health status, chronic illnesses, physical functioning and psychological health, that can influence sleep health that were not included in the analysis. The internal consistency of the technology sub-scales (DSP, TA, and TE) was lower than that observed in the original validation. This difference likely arises from the short length of the scales and restricted variance in an older demographic, which may reduce the accuracy of effect estimates. Future studies could explore more advanced statistical methods as well as have a larger sample to better understand how technology use interacts with sleep health. Furthermore, detailed functional health measures should be included for a holistic association of technology use with sleep health. Longitudinal studies would also help clarify the causal relationships between digital engagement and sleep outcomes in older adults. There are several strengths of this study too. The selection and sampling bias in our study is minimal as participants included in the study are representative of the target population through random sampling in the SNAC project. We also received a good response frequency, indicating increased representation and reduced non-response bias. In this study, a validated instrument was used to measure the outcome, i.e., sleep health, to standardize data collection. In our regression models, we adjusted for potential confounding variables so that the results are valid, reliable, and accurately reflect the true associations among the variables studied.

## Conclusion

Most studies found on technology use and its Most studies found on technology use and its impact on sleep did not focus on older adults. The results of this study, focusing on older adults aged 60 years and above, indicated that being an internet user and spending time using a screen an hour before sleep does not affect sleep negatively, but using mobile phones during wake hours after sleep onset predicted poor sleeping time. Social participation through digital platforms positively affects sleep satisfaction, efficiency, and duration. Additionally, having TA negatively affects sleep satisfaction, timing, and efficiency. Overall, digital technology use does not seem to be negatively associated with sleep health in the older population, with the exception of individuals using mobile phones during wake-time after sleep onset. While these findings provide insight into the relationship between technology use and sleep health, the cross-sectional design and sample limitations mean that causal conclusions cannot be drawn. This study serves as a starting point for future research that can explore these relationships in a wider range of populations and over longer periods. By doing so, we can gain a better understanding of how digital engagement affects sleep health in older adults over time. This knowledge will be helpful in creating targeted interventions to encourage healthy sleep in older adults, considering both the potential risks and benefits of using technology.

## Supplementary Information

Supplementary Material 1.

## Data Availability

The datasets used and/or analyzed during the current study are available from the SNAC-Blekinge principal investigator (Johan Sanmartin Berglund) on reasonable request.
