# Supplementary material for: Examining sleep health and its associations with technology use among older adults in Sweden: insights from a population-based study
Source: BMC Public Health. 2025 Aug 22;25:2896. doi: 10.1186/s12889-025-23894-8 (PMC12372380; doi:10.1186/s12889-025-23894-8)
Supplement: Supplementary file 1 — Supplementary Material 1. [file 12889_2025_23894_MOESM1_ESM.pdf]

Supplementary File explaining the selection process in backward regression as well as the full models for the sensitivity analysis.

Content:

TABLE S1: Variable-selection path of the backward linear regression (Page 1)
Table S2.1-S2.2 : Full model of linear regression for sensitivity analysis (Page 2)
Table S3.1 -3.3 Age-forced linear regression model. (Page3)
Table S4.1-4.5 Variable-selection path of the backward logistic regression (Page 5)
Table S5.1-5.5 Full models of logistic regression for sensitivity analysis. (Page 7)
Table S6: Summary of Logistic regression sensitivity analysis (Page 12)
Table S7: Age forced logistic regression. (Page 12)

Linear regression:

SELECTION PROCESS

Main Linear backward regression

Selection steps and change of Adjusted R2 and Anova significance

Table S1: . Variable-selection path

Step	Variable(s) entered	Variable(s) removed	Method	Adjusted R²	Model *p*(ANOVA)
1	*All predictors forced*(techAnxiety, Tech-to-improve-sleep, Sleep-disorder, Screen ≤ 1 h, Monitor-sleep, Night-phone-use, Living-status, Education, Gender, Tech Enthusiasm, Age-group, Internet-user, Internet-freq, DSP)	—	ENTER	0.060	< .001
2	—	Tech-to-improve-sleep	Backward	0.063	< .001
3	—	Internet-frequency	Backward	0.065	< .001
4	—	Monitor-sleep	Backward	0.066	< .001
5	—	Age-group	Backward	0.067	< .001
6	—	Internet-user	Backward	0.068	< .001
7	—	DSP score	Backward	0.070	< .001
8	—	Gender	Backward	0.070	< .001
9	—	Sleep-disorder	Backward	0.069	< .001

10	—	Night-phone-use	Backward	0.067	< .001
11	—	Living-status	Backward	0.064	< .001

FULL MODELS FOR SENSITIVITY ANALYSIS:

Tables S2.1
MODEL SUMMARY

R	R Square	Adjusted R Square	Std. Error of the Estimate	R Square Change	F Change	df₁	df₂	Sig. F Change	ANOVA Sig.
0.311	0.097	0.060	2.006	0.097	2.643	14	346	.001	0.001

Tables S2.2

Predictor	B	SE	β	t	p	95 % CI for B
**Intercept**	7.157	0.740	—	9.668	< .001	5.701 – 8.613
Age group	−0.137	0.165	−0.048	−0.831	.407	−0.462 – 0.187
Female (vs male)	−0.271	0.230	−0.065	−1.175	.241	−0.723 – 0.182
Living alone	0.196	0.243	0.045	0.806	.421	−0.282 – 0.673
Education	0.253	0.164	0.089	1.545	.123	−0.069 – 0.575
Sleep-disorder diagnosis	−0.410	0.345	−0.062	−1.189	.235	−1.088 – 0.268
Daily internet user	−0.536	0.497	−0.098	−1.077	.282	−1.514 – 0.442
Internet-use frequency ≥ daily	0.187	0.412	0.040	0.454	.650	−0.623 – 0.997
Screen ≤ 1 h before bed	0.408	0.226	0.099	1.808	.071	−0.036 – 0.852
Night-time phone use	−0.676	0.501	−0.071	−1.348	.178	−1.661 – 0.310
Tech to improve sleep	−0.040	0.283	−0.007	−0.140	.888	−0.596 – 0.517
Monitor sleep	−0.330	0.526	−0.033	−0.627	.531	−1.364 – 0.705
Digital-social participation	0.095	0.123	0.076	0.769	.442	−0.147 – 0.336
Tech Enthusiasm	0.189	0.124	0.105	1.526	.128	−0.054 – 0.431
Tech Anxiety	−0.183	0.103	−0.096	−1.781	.076	−0.385 – 0.019

The sensitivity analysis showed that the adjusted R2 in the original backward linear regression (0.064) and forced all predictors is (0.60).

2. Age Forced model

Pure backward dropped every Block-2 variable, leaving only the forced term (age), because in backwards once a variable is removed, it cannot be re-entered into the model. That outcome looks as if technology is irrelevant, even though several tech predictors would still be significant if re-entered. Therefore, to further check the significant variables, we performed stepwise regression.

TABLE S3.1: ***Age-forced model*.**

Model Summary

Model	R	R Square	Adjusted R Square	Std. Error of the Estimate	Change Statistics				
					R Square Change	F Change	df1	df2	Sig. F Change
1	.141	.020	.017	2.05148	.020	7.277	1	359	.007
2	.221	.049	.043	2.02380	.029	10.888	1	358	.001
3	.247	.061	.053	2.01371	.012	4.594	1	357	.033

TABLE S3.2

ANOVA

Model		Sum of Squares	df	Mean Square	F	Sig.
1	Regression	30.626	1	30.626	7.277	.007
	Residual	1510.876	359	4.209		
	Total	1541.501	360			
2	Regression	75.219	2	37.609	9.183	<.001
	Residual	1466.282	358	4.096		
	Total	1541.501	360			

TABLE S3.3

Coefficients

Model		Unstandardized Coefficients		Standardized Coefficients	t	Sig.	95.0% Confidence Interval for B	
		B	Std. Error	Beta			Lower Bound	Upper Bound
1	(Constant)	8.158	.297		27.495	<.001	7.575	8.742
	Age_groups	-.402	.149	-.141	-2.698	.007	-.696	-.109

**2**	**(Constant)**	7.100	.434		16.352	<.001	6.246	7.954
	**Age_groups**	-.311	.150	-.109	-2.076	.039	-.605	-.016
	**techEnthusiasm_23**	.310	.094	.173	3.300	.001	.125	.494
**3**	**(Constant)**	7.820	.547		14.290	<.001	6.744	8.896
	**Age_groups**	-.255	.151	-.089	-1.686	.093	-.552	.042
	**techEnthusiasm_23**	.272	.095	.152	2.856	.005	.085	.459
	**techAnxiety_23**	-.219	.102	-.114	-2.143	.033	-.419	-.018
Dependent Variable: Total sleep score								

Conclusion for linear regression:

The original backward model explains slightly more variance (Adj $R^2 \approx .064$).
Forcing every predictor (full model) shrinks Adj R^2 to .060 – that's expected because Adj R^2 penalises extra terms that add little explanatory power.
Age-forced stepwise drops to Adj $R^2 = .053$, yet both technology attitudes remain significant and in the same directions.
Therefore, the substantive conclusions are robust.

Logistic regression:

SELECTION PROCESS

Satisfaction:

The following charts the backward LR elimination for Sleep Satisfaction. Sequential removals caused only negligible, non-significant fit changes (Step χ^2 range = −0.04 to −1.17; all $p > .55$) and although explanatory power declined (Nagelkerke $R^2 = .114 \rightarrow .107$), the final predictor model remained statistically significant overall (LR $\chi^2 = 30.27$, df = 7, $p < .001$).

Table S4.1

Step	Variable(s) removed*	−2 LL	Nagelkerke R^2	Step χ^2 (df) / p	Overall model χ^2 (df) / p
1	— (full model)	462.461	.114	—	—
2	How often you use internet	462.503	.114	−0.04 (1) / .839	—
3	Living status	462.560	.114	−0.06 (1) / .811	—

4	Education (two dummies)	463.056	.112	−0.50 (2) / .781	—
5	Night-time phone use	463.107	.112	−0.05 (1) / .821	—
6	Tech Enthusiasm	463.194	.112	−0.09 (1) / .768	—
7	Monitor sleep	463.304	.112	−0.11 (1) / .741	—
8	Screen ≤ 1 h before bed	463.506	.111	−0.20 (1) / .653	—
9	Age group	464.674	.107	−1.17 (2) / .558	**30.27 (7) / <.001**

Alertness

The following table illustrates the backward LR elimination path for the Alertness outcome. Successive removals produced only trivial, non-significant changes in model fit (Step χ^2 range = −0.07 to −2.00; all $p > .15$), while the final model remained highly significant overall (LR χ^2 = 43.73, df = 7, $p < .001$) with a modest Nagelkerke R^2 of .149."

Table S4.2

**Step**	**Variable(s) removed**	**−2 LL**	**Nagelkerke R^2**	**Step χ^2 (df) / p**	**Overall model χ^2 (df) / p**
1	— (full model)	460.963	.165	—	—
2	Sleep-disorder	461.035	.165	−0.07 (1) / .788	—
3	Screen ≤ 1 h before bed	461.152	.164	−0.12 (1) / .732	—
4	Tech. to improve sleep	461.321	.164	−0.17 (1) / .681	—
5	Tech Enthusiasm	461.482	.163	−0.16 (1) / .688	—
6	Night-time phone use	461.749	.162	−0.27 (1) / .605	—
7	Tech Anxiety	462.471	.160	−0.72 (1) / .396	—
8	Internet-user (yes/no)	463.303	.157	−0.83 (1) / .362	—
9	Internet-use frequency	463.730	.156	−0.43 (1) / .513	—
10	Living status	465.731	.149	−2.00 (1) / .157	**43.73 (7) / <.001**

Timing

The following table shows the backward LR elimination for the Timing outcome. Successive removals produced only minimal, non-significant changes in model fit (Step χ^2 range = –0.01 to –1.98; all $p > .16$), and the final six-predictor model remained highly significant overall (LR χ^2 = 39.54, df = 6, $p < .001$) with a modest Nagelkerke R^2 of .182."

Table S4.3

Step	Variable(s) removed	−2 LL	Nagelkerke R^2	Step χ^2 (df) / p	Overall model χ^2 (df) / p
1	— (full model)	257.255	.209	—	—
2	Internet-user (yes/no)	257.260	.209	–0.01 (1) / .943	—
3	Sleep-disorder	257.313	.209	–0.05 (1) / .818	—
4	Digital-social-participation score	257.372	.208	–0.06 (1) / .809	—
5	Living status	257.433	.208	–0.06 (1) / .804	—

6	Tech to improve sleep	257.692	.207	–0.26 (1) / .611	—
7	Age group (two dummies)	259.075	.201	–1.38 (2) / .501	—
8	Internet-use frequency	259.824	.198	–0.75 (1) / .387	—
9	Monitor-sleep (yes/no)	261.799	.189	–1.98 (1) / .160	—
10	Tech Enthusiasm	263.522	.182	–1.72 (1) / .189	39.54 (6) / <.001

Efficiency

The following charts the backward LR elimination for Sleep Efficiency. Sequential removals caused only negligible, non-significant fit changes (Step χ^2 range = 0.00 to −4.61; all $p > .10$), and although explanatory power declined (Nagelkerke R^2 = .075 → .044), the final predictor model remained statistically significant overall (LR χ^2 = 12.12, df = 3, $p = .007$).

Table S4.4

Step	Variable(s) removed	−2 LL	Nagelkerke R^2	Step χ^2 (df) / p	Overall model χ^2 (df) / p
1	— (full model)	462.246	.075	—	—
2	Living status	462.246	.075	0.00 (1) / 1.000	—
3	Monitor sleep	462.247	.075	–0.00 (1) / .978	—
4	Internet-use frequency	462.248	.075	–0.00 (1) / .968	—
5	Tech to improve sleep	462.271	.075	–0.02 (1) / .881	—
6	Sleep-disorder	462.309	.075	–0.04 (1) / .845	—
7	Gender	462.373	.075	–0.06 (1) / .800	—
8	Tech Enthusiasm	462.480	.074	–0.11 (1) / .744	—
9	Internet-user (yes/no)	462.649	.074	–0.17 (1) / .682	—
10	Age group (2 dummies)	464.516	.067	–1.87 (2) / .393	—
11	Screen ≤ 1 h before bed	465.372	.064	–0.86 (1) / .355	—
12	Night-time phone use	466.244	.061	–0.87 (1) / .350	—
13	Education (2 dummies)	470.849	.044	–4.61 (2) / .100	12.12 (3) / .007

Duration

The following table depicts the backward LR elimination for the Duration outcome. Each successive removal caused only small, non-significant reductions in fit (Step χ^2 range = −0.02 to −2.37; all $p > .27$), and the final four-predictor model remained statistically significant overall (LR χ^2 = 27.33, df = 4, $p = .003$) with a modest Nagelkerke R^2 of .103.

Table S4.5

**Step**	**Variable(s) removed**	**−2 LL**	**Nagelkerke R^2**	**Step χ^2 (df) / p**	**Overall model χ^2 (df) / p**
1	— (full model)	404.356	.126	—	—
2	Living status	404.377	.126	−0.02 (1) / .885	—
3	Tech-to-improve-sleep	404.396	.126	−0.02 (1) / .889	—
4	Monitor sleep	404.432	.126	−0.04 (1) / .850	—
5	Tech Enthusiasm	404.607	.125	−0.18 (1) / .676	—
6	Age group (2 dummies)	405.757	.121	−1.15 (2) / .563	—
7	Internet-use frequency	406.229	.119	−0.47 (1) / .492	—

8	Internet-user (yes/no)	406.466	.119	−0.24 (1) / .626	—
9	Sleep-disorder	407.138	.116	−0.67 (1) / .412	—
10	Education (2 dummies)	409.507	.108	−2.37 (2) / .306	—
11	Night-phone-screen	410.709	.103	−1.20 (1) / .273	27.33 (4) / .003

FULL MODELS FOR SENSITIVITY ANALYSIS:

Satisfaction:

Table S5.1

Predictor	B	SE	Wald χ^2	p	OR (95 % CI)
Age group (*60–74 y*)					
75–85 y	0.023	0.265	0.01	.931	1.02 (0.61–1.72)
≥ 85 y	–0.319	0.353	0.81	.367	0.73 (0.36–1.45)
Female (vs male)	–0.732	0.245	8.93	.003	0.48 (0.30–0.78)
Living alone	–0.060	0.258	0.05	.817	0.94 (0.57–1.56)
Education (*primary*)					
Secondary	0.129	0.313	0.17	.680	1.14 (0.62–2.10)
Higher	–0.041	0.351	0.01	.907	0.96 (0.48–1.91)
Sleep-disorder diagnosis	–0.758	0.358	4.49	.034	0.47 (0.23–0.94)
Daily internet use	–0.886	0.526	2.84	.092	0.41 (0.15–1.16)
Internet-use frequency ≥ daily	–0.087	0.430	0.04	.839	0.92 (0.39–2.13)
Screen ≤ 1 h before bed	0.396	0.238	2.27	.132	1.49 (0.89–2.49)
Night-time phone use	0.167	0.537	0.10	.755	1.18 (0.41–3.39)
Monitor sleep	0.229	0.584	0.15	.696	1.26 (0.40–3.95)
Tech to improve sleep	–0.573	0.298	3.70	.055	0.56 (0.31–1.01)
Digital-social participation	0.259	0.132	3.86	.050	1.30 (1.00–1.68)
Tech Enthusiasm	0.040	0.131	0.09	.761	1.04 (0.81–1.35)
Tech Anxiety	–0.207	0.111	3.47	.063	0.81 (0.65–1.01)
Constant	1.622	0.662	6.00	.014	5.06

Model fit:

- −2 Log Likelihood = 462.461
- Nagelkerke R^2 = .114
- Hosmer–Lemeshow χ^2(df) = 8.759 (8) *p* = 0.363

Alertness

Table S5.2

Predictor	B	SE	Wald χ^2	p	OR (95 % CI)
Age group (*60–74 y*)					
75–85 y	-0.669	0.267	6.28	.012	0.51 (0.30–0.86)
≥ 85 y	-0.852	0.355	5.77	.016	0.43 (0.21–0.86)
Female (vs male)	0.799	0.249	10.33	.001	2.22 (1.37–3.62)
Living alone	0.341	0.262	1.69	.193	1.41 (0.84–2.35)
Education (*Primary*)					
Secondary	0.129	0.310	0.17	.680	1.38 (0.75–2.54)
Higher	0.742	0.348	4.53	.033	2.10 (1.06–4.16)
Sleep-disorder diagnosis	-0.097	0.361	0.07	.788	0.91 (0.45–1.84)
Daily internet use	-0.477	0.531	0.81	.369	0.62 (0.22–1.76)
Internet-use frequency ≥ daily	0.370	0.431	0.74	.391	1.45 (0.62–3.37)
Screen ≤ 1 h before bed	0.081	0.238	0.12	.732	1.11 (0.70–1.73)
Night-time phone use	-0.266	0.566	0.22	.639	0.77 (0.25–2.32)
Monitor sleep	-1.811	0.635	8.14	.004	0.16 (0.05–0.57)
Tech to improve sleep	-0.124	0.304	0.17	.683	0.88 (0.49–1.60)
Digital-social participation	0.110	0.130	0.72	.397	1.12 (0.87–1.44)
Tech Enthusiasm	0.052	0.131	0.16	.761	1.05 (0.81–1.36)
Tech Anxiety	-0.085	0.110	0.60	.439	0.92 (0.74–1.14)
Constant	-0.412	0.650	0.40	.526	0.66

Model fit:

- −2 Log Likelihood = 460.963
- Nagelkerke R^2 = 0.165
- Hosmer–Lemeshow χ^2(df) = 5.808 (8) *p* = 0.669

Timing

Table S5.3

Predictor	B	SE	Wald χ^2	p	OR (95 % CI)
Age group (*60–74 y*)					
75–85 y	0.353	0.403	0.77	.380	1.42 (0.65 – 3.13)
≥ 85 y	–0.024	0.499	0.00	.962	0.98 (0.37 – 2.60)
Female (vs male)	–1.001	0.370	7.31	.007	0.37 (0.18 – 0.76)
Living alone	0.094	0.362	0.07	.796	1.10 (0.54 – 2.24)
Education (Primary)					

Secondary	–0.654	0.426	2.36	.124	0.52 (0.23 – 1.20)
Higher	0.281	0.535	0.28	.599	1.32 (0.46 – 3.78)
Sleep-disorder diagnosis	0.127	0.553	0.05	.819	1.14 (0.38 – 3.36)
Daily internet use	0.050	0.702	0.01	.943	1.05 (0.27 – 4.17)
Internet-use frequency ≥ daily	0.191	0.574	0.11	.739	1.21 (0.39 – 3.73)
Screen ≤ 1 h before bed	1.018	0.369	7.63	.006	2.77 (1.34 – 5.70)
Night-time phone use	–2.043	0.627	10.63	.001	0.13 (0.04 – 0.44)
Monitor sleep	–1.093	0.722	2.29	.130	0.34 (0.08 – 1.38)
Tech to improve sleep	–0.190	0.402	0.22	.636	0.83 (0.38 – 1.82)
Digital-social participation	0.034	0.198	0.03	.863	1.04 (0.70 – 1.52)
Tech Enthusiasm	0.133	0.196	0.46	.498	1.14 (0.78 – 1.68)
Tech Anxiety	–0.319	0.158	4.05	.044	0.73 (0.53 – 0.99)
Constant	2.766	0.941	8.64	.003	15.90

Model fit:

- −2 Log Likelihood = 257.255
- Nagelkerke R^2 = 0.209
- Hosmer–Lemeshow χ^2(df) = 8.787 (8) p = 0.361

Efficiency

Table S5.4

Predictor	B	SE	Wald χ^2	p	OR (95 % CI)
Age group (*60–74 y*)					
75–85 y	0.086	0.264	0.11	.743	1.09 (0.65 – 1.83)
≥ 85 y	0.469	0.357	1.72	.189	1.60 (0.79 – 3.22)
Female (vs male)	0.064	0.244	0.07	.792	1.07 (0.66 – 1.72)
Living alone	0.000	0.263	0.00	1.000	1.00 (0.60 – 1.67)
Education (*Primary*)					
Secondary	−0.232	0.317	0.53	.465	0.79 (0.43 – 1.48)
Higher	0.343	0.347	0.98	.322	1.41 (0.72 – 2.78)
Sleep-disorder diagnosis	0.072	0.363	0.04	.843	1.08 (0.53 – 2.19)
Daily internet use	0.240	0.552	0.19	.663	1.27 (0.43 – 3.75)
Internet-use frequency ≥ daily	−0.018	0.442	0.00	.968	0.98 (0.41 – 2.34)
Screen ≤ 1 h before bed	0.214	0.236	0.82	.365	1.24 (0.78 – 1.97)
Night-time phone use	−0.508	0.547	0.86	.353	0.60 (0.21 – 1.76)
Monitor sleep	0.015	0.539	0.00	.978	1.02 (0.35 – 2.92)
Tech to improve sleep	−0.046	0.305	0.02	.880	0.96 (0.53 – 1.74)
Digital-social participation	0.115	0.129	0.81	.369	1.12 (0.87 – 1.44)
Tech Enthusiasm	0.049	0.130	0.14	.705	1.05 (0.82 – 1.35)

Tech Anxiety	−0.239	0.110	4.70	.030	0.79 (0.63 – 0.98)
Constant	−0.669	0.655	1.04	.307	0.51

Model fit:

- −2 Log Likelihood = 462.246
- Nagelkerke $R^2 = 0.075$
- Hosmer–Lemeshow χ^2(df) = 8.216 (8) $p = 0.413$

Duration:

Table S5.5

Predictors	B	SE	Wald χ^2	*p*	OR (95 % CI)
Age group (60–74 y)					
75–85 y	–0.290	0.299	0.95	.331	0.75 (0.42–1.34)
85+ y	–0.326	0.387	0.71	.400	0.72 (0.34–1.54)
Female (vs. male)	–0.848	0.271	9.83	.002	0.43 (0.25–0.73)
Living alone (vs. co-habiting)	0.040	0.278	0.02	.885	1.04 (0.60–1.79)
Education (basic)					
Secondary	–0.249	0.328	0.57	.449	0.78 (0.41–1.49)
Higher	0.132	0.383	0.12	.730	1.14 (0.54–2.42)
Sleep-disorder diagnosis	–0.316	0.385	0.68	.411	0.73 (0.34–1.55)
Daily internet use (yes)	–0.393	0.543	0.53	.469	0.68 (0.23–1.96)
Internet-use frequency	0.279	0.444	0.40	.529	1.32 (0.55–3.16)

Screen ≤ 1 h before bed (yes)	0.396	0.263	2.27	.132	1.49 (0.89–2.49)
Phone use during night awakenings (yes)	–0.777	0.553	1.98	.160	0.46 (0.16–1.36)
Monitor sleep (yes)	0.122	0.681	0.03	.858	1.13 (0.30–4.29)
Tech to improve sleep (yes)	0.051	0.324	0.03	.875	1.05 (0.56–1.98)
Digital-social participation	0.135	0.145	0.86	.353	1.14 (0.86–1.52)
Tech Enthusiasm	0.057	0.145	0.15	.694	1.06 (0.80–1.41)
Tech Anxiety	–0.153	0.119	1.65	.199	0.86 (0.68–1.08)
Constant	1.660	0.713	5.42	.020	5.26

Model fit:

- −2 Log Likelihood = 404.356
- Nagelkerke R^2 = 0.126
    - Hosmer–Lemeshow χ^2(df) =5.588 (8) p = 0.693

Summary Logistic regression sensitivity analysis:

Table S6

	**Backward**	**Full**
**Satisfaction**	.107	.114
**Alertness**	0.149	0.165

**Timing**	.182	0.209
**Efficiency**	.044	0.075
**Duration**	.103	0.126

Forced age, while backward regression of all the predictors so that significant variables survive:

TABLE S7

Outcome	Backward model(primary)	Age-forced model(age in Block 1, backward Block 2)	Full model(all predictors forced)
**Alertness**	0.149	0.149	0.165
**Duration**	0.103	0.090	0.126

The above table (S7) shows that for Alertness, forcing age leaves model fit unchanged ($R^2 = 0.149$); adding back every discarded predictor raises R^2 by only 0.016.

For Duration, forcing age lowers fit slightly (0.103 → 0.090), while the full model gains just 0.023 over the primary solution. Thus, neither the inclusion of age nor the retention of all low-information predictors materially improves explanatory power, confirming the robustness of the backward models.

Only these two outcomes were checked because we found a significant difference between them among age groups.
